# Clinicopathological analysis and outcome of primary mediastinal malignancies — A report of 91 cases from a single institute

**DOI:** 10.4103/1817-1737.53354

**Published:** 2009

**Authors:** Biswajit Dubashi, Sanju Cyriac, Sagar Gnana Tenali

**Affiliations:** *Department of Medical Oncology, Cancer Institute (WIA), Chennai, India*

**Keywords:** Germ cell tumor, lymphoma, primary mediastinal tumor, thymoma

## Abstract

**BACKGROUND:**

Primary mediastinal malignancies are uncommon. They can originate from any mediastinal organ or tissue but most commonly arise from thymic, neurogenic, lymphatic, germinal or mesenchymal tissues.

**OBJECTIVES:**

The aim of this study was to review the clinical presentations, diagnostic methods adopted, the histologies and the treatment outcomes of this rare subset of tumors.

**MATERIALS AND METHODS:**

Case records of 91 patients in the period 1993-2006 at our institute were retrospectively analyzed. Patients with primary mediastinal mass and supraclavicular nodes were included for the analysis. Patients with primary, extrathoracic disease of the lung and peripheral adenopathy were excluded. Actuarial method was used for calculating the disease-free survival and overall survival.

**RESULTS:**

Primary mediastinal tumors were seen commonly in males with mean age of 37.48 ± 17.04 years. As many as 97% of patients were symptomatic at presentation. Superior venacaval obstruction (SVCO) was seen in 28% of the patients. As many as 50% of the patients were diagnosed by a fine-needle aspiration or Trucut biopsy, while 28% of the patients required thoracotomy for a diagnosis. Majority of the tumors had anterior mediastinal presentation. Pleural effusion was seen in 20% of the patients, but diagnosis was obtained in only 1%. In adults, thymoma (39%), lymphoma (30%) and germ cell tumor (15%) were the common tumors. In the pediatric population, lymphoma, PNET and neuroblastoma were the common tumors. The 5-year DFS and OS are 50% and 55%, respectively.

**CONCLUSION:**

Primary mediastinal tumors are a challenge to the treating physician because of their unique presentation in the form of medical emergencies, like superior venacaval obstruction and stridor. Diagnosis may require invasive procedures like thoracotomy. Treatment and outcome depend on the histologic subtypes.

Primary mediastinal malignancies are rare tumors.[1,2] They are most often seen in the third to fifth decades of life. Symptomatic presentation is seen in 60% of the patients.[3] They can present as an oncological emergency in the form of superior venacaval obstruction (SVCO) syndrome and stridor. Thoracotomy may be required for a diagnosis. They may be associated with various paraneoplastic syndromes. Thymoma, germ cell tumor, lymphoma and neurogenic tumors are the common histologic subtypes. The incidence of various subtypes differs according to the compartment.[4]

The aim of this study was to review the clinical presentations, diagnostic methods adopted, the histologies and the treatment outcomes of this rare subset of tumors.

## Materials and Methods

Case records of all patients diagnosed with primary malignant mediastinal tumors between 1^st^ January 1993 and 31^st^ December 2006 were retrospectively reviewed from the tumor registry of the Cancer Institute hospital, Chennai. Patients with primary mediastinal presentation were included for the study. Patients with primary tumors of the thyroid, parathyroid, airway, esophagus and lung were excluded. Clearances from the ethical and research committees were taken prior to the analysis. The age limit for pediatric patients was 14 years.

The data were entered in Microsoft Excel and analyzed using the statistical package for social sciences (SPSS Inc., for personal computer version 10 software. Data was expressed in text and Tables as mean ± standard deviation. Actuarial method was used for calculating the disease-free survival and overall survival.

## Results

A total of 91 cases were analyzed. The mean age at diagnosis was 37.48 ± 17.04 years (range, 1–76 years). There were 9 pediatric patients and 842 adult patients. There was a male predominance, with a male-to-female ratio of 2.8:1. The mean duration of symptoms was 125 days (range, 14–720 days). As many as 97% of the patients were symptomatic at presentation, with cough (83.5%), chest pain (56%) and dyspnea (75.8%) as predominant symptoms. As many as 24% of the patients were chronic smokers. Supraclavicular node (21.9%), pleural effusion (20.8%), SVCO (28.5%) and stridor (2.2%) were the common signs observed in this subset. Five patients presented with paraneoplastic syndromes, of which 4 patients had myasthenia gravis and 1 patient had pure red cell aplasia.

The various histologies encountered are listed in Table 1. Around 50% of the patients were diagnosed by fine-needle aspiration cytology (FNAC) or Trucut biopsy, while 28% of the patients required thoracotomy for a diagnosis, predominantly the thymic tumors [Table 2]. Eight patients required 3 or more attempts for tissue diagnosis. Tumors requiring multiple attempts included thymoma and lymphoma. Twenty-five patients required thoracotomy for a diagnosis. The predominant tumors which required thoracotomy included thymoma, 11 (44%) patients; Thymic carcinoma 2 (8%); and lymphomas 4 (16%) patients. Pleural effusion was seen in 42% of the patients, but diagnosis was obtained in only 1%.

**Table 1:** Histopathologic distribution of primary mediastinal tumors

Histopathology	*n* = 91 (%)
Thymoma	28 (30.7)
Thymic carcinoma	5 (5.4)
Non-Hodgkin lymphoma	13 (14.3)
Hodgkin disease	6 (6.5)
Lymphoblastic lymphoma	9 (9.8)
Seminoma	6 (6.5)
Non-seminomatous germ cell tumor	8 (8.8)
Poorly differentiated carcinoma	8 (8.8)
Primary neurectodermal tumor	4 (4.4)
Neuroblastoma	2 (2.2)
Sarcoma	2 (2.2)

**Table 2:** Clinical presentation and outcome of different histological subtypes of primary malignant mediastinal tumors

Characteristics	Thymic tumors	Lymphoma	Seminoma	NSGCT	Overall
n	33	28	6	8	91
Mean age (years)	50.04 ± 14.8	31.57 ± 12.1	45	28	37.48 ± 17.04
Male:Female	1.8:1	3.6:1	Males	Males	2.8:1
SVCO*	4	12	3	0	26
Thoracotomy for diagnosis	11	4	3	0	25
Treatment modalities					
Chemotherapy	4	3	3	4	27
Chemotherapy+RT†	2	25	3	1	31
Surgery	8				8
Surgery+radiotherapy	8				10
Chemotherapy+surgery	2			3	2
Radiotherapy	2				2
Surgery+Chemo+RT	4				7
Best supportive care	3				4
DFS‡ % (years)	63 (2)	57 (2)	80 (5)	33 (5)	50 (5)
OS§ %(years)	70 (2)	58 (2)	80 (5)	46 (5)	55 (5)

* - Superior venacaval obstruction;

† - Radiotherapy;

‡ - Disease-free survival;

§ - Overall survival

**Figure 1:**
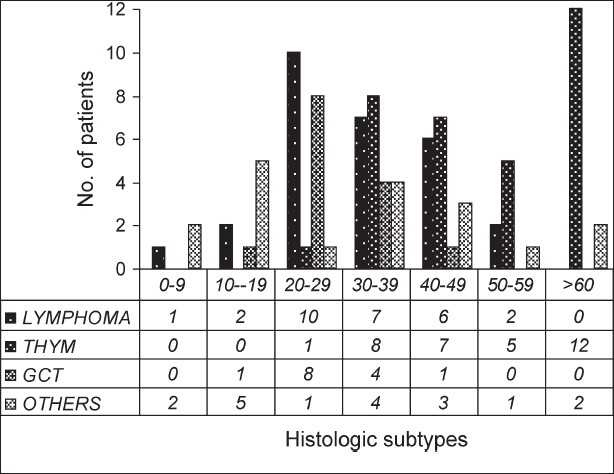
Age distribution of primary mediastinal tumors

Majority of the tumors (93.6%) had anterior mediastinal presentation. Posterior mediastinal tumors were seen in 6.4% of the patients, with primary neurectodermal tumors (PNET) and neurogenic tumors being common. In pediatric population, lymphoma, PNET and neuroblastoma were the common tumors. The age distribution of various primary mediastinal tumors is demonstrated in Figure 1.

Emergency steroid was initiated in 11.8% of the patients. Three patients required emergency radiotherapy. Surgery, chemotherapy and radiotherapy were the various treatment modalities used depending on the histologic subtypes [Table 2].

Patients with thymoma were elderly, with mean age of 50.04 ± 14.80 years and male predominance. Five patients had paraneoplastic syndrome at presentation. Myasthenia gravis was seen in 4 patients; and pure red cell aplasia, in 1 patient. The 2-year disease-free survival (DFS) and overall survival (OS) were 63% and 70%, respectively.

The mean age of patients with germ cell tumor was 28 ± 7.36 years. As many as 43% of the patients had seminoma, and non-seminomatous germ cell tumor (NSGCT) was seen in 57% of the patients. Platinum-based treatment was the primary treatment in 84% of the patients. The 5-year DFS and OS in seminoma were 80%, respectively; while in NSGCT, these were 33% and 46%, respectively.

The mean age of patients with lymphomas was 31.57 ± 12.1 years. Non-Hodgkin lymphoma (46%), Hodgkin disease (21%) and lymphoblastic lymphoma (32%) were the common histologies. SVCO was seen in 42% of the patients. As many as 14% of the patients underwent thoracotomy for a diagnosis. The 2-year DFS and OS were 57% and 58%, respectively [Table 2].

The median duration of follow-up of all primary mediastinal tumors was 7 years. The 5-year disease-free survival and overall survival were 50% and 55%, respectively.

## Discussion

Primary mediastinal tumors are uncommon, representing approximately 3% of the tumors within the chest.[1,2] As many as 25% to 49% of these lesions are malignant.[1] Our study represents 91 patients, 0.1% of the patients within our registry, which represents a large series containing exclusively primary malignant mediastinal neoplasms.

As many as 97% of our patients were symptomatic at presentation. Davis *et al.* found that 85% of the patients with malignancy were symptomatic; only 46% of the patients with benign neoplasms had identifiable complaints.[5]

CT-guided percutaneous biopsy is standard in the initial evaluation of mediastinal masses.[6] In our study, 54.8% of the patients were diagnosed by percutaneous biopsy. As many as 27.4% of our patients required thoracotomy for a diagnosis, which was slightly higher in our study.[6]

The ratio of adult-to-pediatric patients with primary mediastinal tumors was 9.3:1; whereas in the study by Azarow *et al.,* it was 3.1:1.[3] Age distribution revealed lymphoma and germ cell tumor occurring in the period from third to fourth decade of life, while thymic neoplasms predominantly occurred in the fifth decade.

The most common tumor in our series was thymic neoplasm, which constituted 36.1%, followed by lymphoma (30.6%) and germ cell tumor (15.3%). Azarow *et al.* reported the Walter Reed experience: Of the 254 primary mediastinal masses reported, thymic malignancies were most common.[3] In the study by Roy Temes, lymphoma was the most common histology, seen in 55% of the patients, two thirds constituted by non-Hodgkin lymphoma.[4]

Germ cell tumors represented 3 to 14% of primary malignant mediastinal lesions in various studies.[1,5] Seminoma was seen in 6 (43%) patients; while NSGCT, in 8 (57%). In various studies, seminoma was found to be the most common malignant germ cell tumor of the mediastinum and has been reported to occur in 21% to 50% of the patients with malignant mediastinal germ cell tumors.[2–5] The 5-year survival in patients with seminoma and NSGCT in our study was 80% and 46%, respectively. Other studies have reported overall 5-year survivals of 45% for NSGCT and 58% to 82% for seminomas.[4]

Rubush *et al.* found thymoma in 59% of the 61 patients reported.[1] There were no cases in the pediatric population. Study by Azarow *et al.* found thymoma in 34% of the pediatric patients.[3] Compared with various studies which reported a survival of 65% to 79% at 5 years,[7] our study showed a lower 5-year survival, viz., 48%, as majority (71.4%) of our patients had advanced presentation.

Neurogenic tumors were predominantly seen in the pediatric population. We had 4 patients with PNET occurring primarily in the mediastinum.

Poorly differentiated carcinoma was seen in 8.8% of the patients. Studies have reported primary carcinoma of the mediastinum in 7% to 30% of tumors.[4] Sarcomas were seen in only 2.2% of the patients. Other studies have reported sarcomas in 2% to 8% of primary malignant mediastinal tumors.[1,2,4,6]

## Conclusion

This study is unique as it included only primary malignant mediastinal tumors. In our study, younger population predominated, and an invasive procedure like thoracotomy was often required for diagnosis. Thymic tumors, lymphomas and germ cell tumors were the predominant histologies. Survival was best for seminoma and the worst for non-seminomatous germ cell tumors. The 5-year overall survival was poor (55%) in spite of multimodality treatment, which emphasizes the need for further advances in treatment.

## References

[1] Rubush JL, Gardner IR, Boyd WC, Ehrenhaft JL (1973). Mediastinal tumors: Review of 186 cases. J Thorac Cardiovasc Surg.

[2] Wongsangiem M, Tangthangtham A (1996). Primary tumors of the mediastinum: 190 cases analysis (1975–1995). J Med Assoc Thai.

[3] Azarow KS, Pearl RH, Zurcher R, Edwards FH, Cohen AJ (1993). Primary mediastinal masses: A comparison of adult and pediatric populations. J Thorac Cardiovasc Surg.

[4] Temes R, Chavez T, Mapel D, Ketai L, Crowell R, Key C (1999). Primary mediastinal malignancies: Findings in 219 patients. West J Med.

[5] Davis RD, Oldham HN, Sabiston DC, Sabiston DC (1989). The Mediastinum. Surgery of the chest.

[6] Morrissey B, Adams H, Gibbs AR, Crane MD (1993). Percutaneous needle biopsy of the mediastinum: Review of 94 procedures. Thorax.

[7] Blumberg D, Port JL, Weksler B, Delgado R, Rosai J, Bains MS (1995). Thymoma: A multivariant analysis of factors predicting survival. Ann Thorac Surg.

